# The importance of crafting a good introduction to scholarly research: strategies for creating an effective and impactful opening statement

**DOI:** 10.5116/ijme.6499.82af

**Published:** 2023-06-29

**Authors:** Mohsen Tavakol, David O'Brien

**Affiliations:** 1 Medical Education Centre, School of Medicine, The University of Nottingham, UK

**Keywords:** Effective Introduction, Introduction structure, scholarly research, impactful opening statement

## Introduction

The introduction section is arguably one of the most critical elements of a written piece of research work, often setting the tone for the remainder of any dissertation or research article. The primary purpose of an introduction is to provide the reader with a clear understanding of the research question, in addition to the scope, rationale, aims and objectives of the study. This ensures the reader can more easily comprehend the context of the research, which will consequently help them better interpret and evaluate the study results. One could liken an introduction to a trailer for a movie, where the plot of the film (the research topic) is introduced by setting the scene (outlining the significance of the topic) and enticing you to watch the full movie (understanding the research and its importance).

Despite this, our experience suggests that students frequently pay insufficient attention to the introduction section of their dissertation or omit elements which we consider essential to address. This editorial aims to help researchers appreciate the importance of a comprehensive dissertation introduction in medical education research and learn how to effectively manage this key section of their work.  Although it focuses purely on the introduction section of a written research submission, readers interested in learning more about the other primary steps of the research process are encouraged to read AMEE Guide No. 90 ^1^^,^^2^ textbooks on research methods and both consult and seek constructive feedback from colleagues with expertise in research methods and writing for publication.

Here we aim to provide the reader with a simple structure of how best to construct the introduction for a dissertation and recommend that this should typically include the following essential components and principles.

### Background to the research topic

The purpose of providing background information in an introduction is to supply the context and other essential information concerning the research topic, and thus allow the reader to understand the significance of the specific research question and where it sits within the broader field of study. This aids the reader to better understand how the research question contributes to the existing body of knowledge and why it is, necessary to investigate this specific aspect further. For example, suppose the study concerns the effectiveness of simulation-based training in medical education. In this case, the broader field of the study may include relevant areas such as medical simulation, medical education research, health care education, standardised patients, simulation-based training, and curriculum development based on simulation training. After providing the reader with an understanding of the context and relevance of the topic of interest, the researcher must then establish a theoretical or conceptual framework. This underpins the study topic in order that the reader can understand how any research questions and objectives are formulated. It is important to distinguish between these two frameworks. A theoretical framework describes the rationale for applying a particular theory to provide support and structure for the topic being studied. In the absence of an applicable theory, a conceptual framework substantiates the significance of a particular problem, context or phenomenon within a specific area of the study by illustrating its relevance and connection to research topic.^3^ A conceptual framework highlights the importance of a research topic by showing how it relates to the larger body of knowledge in a particular field. Here is an example to demonstrate the use of a theoretical framework in a research context.

When considering Social Cognitive Theory (SCT), one of the key constructs is self-efficacy, as described by Albert Bandura,^4^ and refers to the belief that a person has it within their own ability to accomplish a specific task successfully. This is not related to what a person does, but more how they perceive their ability to use these skills. So, based on this construct of self-efficacy, a researcher may formulate a research hypothesis; that examiners with higher self-efficacy in OSCEs will demonstrate improved performance in subsequent exams compared to those with lower self-efficacy. Now the researcher is in a position to identify the fundamental concepts of the research, i.e., self-efficacy (personal factors), examiner performance (behavioural factors) and examination conditions and examiner scaffolding support (environmental factors). Identifying key concepts helps the researcher find the relationship between these, and develop appropriate research questions, e.g., 1) How does an examiner's self-efficacy in OSCEs affect their ability to assess students in subsequent exams? 2) How does the support provided to examiners and exam conditions influence the link between self-efficacy and examiner performance in OSCEs? 3) Do examiners with high self-efficacy provide fairer scores than those with low self-efficacy in OSCEs? By having a theoretical framework, researchers can establish a foundation for their research and provide a clear picture of the relationship between the key concepts involved in the study. Researchers must also provide any conceptual and operational definitions for key concepts or variables that will be used in the study. Clearly defining key concepts and variables in the background section of a dissertation can also help establish the significance of the research question and its relevance to the broader field of study. As the name implies, a conceptual definition refers to a variable's meaning in a conceptual, abstract, or theoretical sense. Conceptual definitions are often used to describe concepts which cannot be directly measured, such as active learning, rote learning, inter-professional learning, inter-professional education, or constructs such as clinical performance. Conversely, operational definitions define the steps researchers must take in order to collect data to measure a phenomenon or concept.^5^ For example, clinical performance can be considered a conceptual construct but may also be defined operationally as the ability of students to pass 12 out of 16 stations of an OSCE. The researcher having already pre-specified specific the criteria for classifying students as pass/fail in order to determine the ability of students to perform clinically. This operational definition provides a clear method for evaluating and measuring student ability, which can then be used to give feedback and guide further learning or to establish clear expectations for students and provide a basis for evaluating and assessing their performance. In general, it can be beneficial for medical education programs to define aspects such as clinical performance operationally in this way in rather than conceptually, especially if there is a need to ensure that students meet a required standard of competence and are prepared for the demands of real-world clinical practice. These definitions can also then be used to establish the methods and criteria by which the variables of the study will subsequently be measured or altered.

### Citing the existing literature to support the research aim

A literature review is the process of critically evaluating existing research and utilising it to inform and guide the research proposal under investigation. Taking this approach enables researchers to ensure that their research is not only grounded in, but also contributes meaningfully to, any existing knowledge as a whole. Critically reviewing the literature provides evidence and justification for any research and is essential when formulating a hypothesis, question, or study objectives. In addition, and perhaps most importantly, it helps identify any gaps or inconsistencies in the existing knowledge base. Determining the knowledge gap is critical in justifying the necessity for our research and advancing knowledge. A comprehensive literature review also helps establish the theoretical or conceptual frameworks to ground any subsequent research, providing researchers with guidance and direction on how best to conduct their future studies. Understanding from the literature what has worked previously and what may pose challenges or limitations assists researchers when exploring the best methods and techniques for answering new research questions. To clarify, consider a hypothetical study in which researchers wish to examine the effectiveness of a specific educational intervention in medical students to improve patient safety. Based on the existing literature, let's assume that researchers learned that most studies had only focused on short-term outcomes rather than long-term ones. The long-term effects of any intervention in medical students on patient safety therefore remain uncertain. Researchers may therefore wish to consider conducting longitudinal studies months after interventions have been carried out, rather than simply repeating research based on short-term outcomes, in order to address the current knowledge gap. A review of existing literature may highlight hitherto previously unconsidered logistical difficulties in conducting longitudinal studies in this area that the researcher may need to be aware of.

### Stating the significance of the research

More than simply reporting the existing research, one of the key objectives in any literature review is to summarise and synthesise existing research on the intended topic in order to analyse the significance of the research in question. In this process, diverse ideas can be merged to form fresh new perspectives. Any gaps, limitations, or controversies in medical education can be identified, and potential future benefits and implications of the proposed research explained to the reader. Based on any potential impact or perceived importance, the introduction provides an excellent opportunity for the researcher to affirm the significance of the research study and why it should be conducted.

By way of an example, the significance of a study concerning feedback given to examiners for Objective Structured Clinical Examinations (OSCEs) is used to illustrate this point further. The potential significance of this research lies in improving the validity and reliability of OSCE scores in medical education. As a result of reviewing different types of feedback given to examiners, the research may assist in identifying the most effective strategies for improving the quality of OSCEs in medical education. By providing new insights into how feedback can improve the reliability and validity of OSCE results, the research could also contribute to the broader knowledge of assessment in general. This may result in the development of more accurate and robust medical education assessments, which in turn may potentially enhance delivery of healthcare and improve patient outcomes and safety. It may also address the current challenges and gaps in medical education assessment by providing evidence-based approaches for improving OSCE quality.

### Formulating Research Questions and Objectives

Researchers formulate research questions and objectives based on the topic they are seeking to address. As noted previously, these will have already been derived as a result of a comprehensive literature review of any existing knowledge and based on a theoretical or conceptual framework. Furthermore, in medical education, the literature review provides researchers with the opportunity to formulate new research questions or research objectives to address any gaps or limitations in the existing literature and add something new to the current body of knowledge. Research questions and objectives should be stated clearly, being both specific, and measurable. These should then guide the subsequent selection of appropriate research methods, data collection and any subsequent analytical process. Clear, focused, and rigorous research questions and objectives will ensure the study is well-designed and make a valuable contribution to the existing body of knowledge.

Qualitative research questions should be open-ended and exploratory rather than focused on a specific hypothesis or proposition. It is common for qualitative studies to focus on understanding how and why certain phenomena occur, rather than simply describing what has occurred. These should be formulated to elicit rich, detailed, and context-specific data that can provide insights into the experiences, perspectives, and meanings of the participants. In contrast, quantitative research questions are more specific and are designed to test a particular hypothesis or relationship. In medical education, it is imperative to emphasise the importance of both qualitative and quantitative research questions when it comes to generating new knowledge. Combining both quantitative and qualitative research methods (mixed methods) can be particularly powerful in providing a more comprehensive understanding of any phenomena under study. Assume again that we are examining the effectiveness of feedback on the performance of medical students and adopt a mixed-methods approach using a combination of qualitative and quantitative research methods. A quantitative research question may be, what is the impact of feedback on the performance of medical students as measured by OSCE mark? How the experience of receiving feedback on performance contributes to the future professional development of medical students is a more qualitative research question. This combination of quantitative and qualitative research questions will provide an in depth understanding of the effectiveness of feedback on medical student performance. It is important to note that in qualitative research methods particularly, there can be a wide variety of research question types. For example, grounded theory researchers may ask so-called "process questions", such as 'how do students interpret and use the feedback they are given?' Phenomenologists, on the other hand, are concerned with lived experience of research subjects and frequently ask questions looking to understand the "meaning" of any such experience, often aiming to attribute feelings to this experience, for example, ‘how do students feel when they receive feedback?’ Ethnographers look to understand how culture contributes to an experience, and may ask more "descriptive questions"^5^ for example, ‘how does the culture within a specific medical school affect students receiving feedback on their performance?’

For ease of reference, the key points we recommended are considered in any dissertation introduction are summarised below:

1.       Set the context for the research

2.       Establish a theoretical or conceptual framework to support your study

3.       Define key variables both conceptually and theoretically

4.       Critically appraise relevant papers during the literature review

5.       Review previous studies to identify and define the knowledge gap by assessing what has already been studied and what areas remain unexplored

6.       Clearly articulate the rationale behind your study, emphasising its importance in the intended field

7.       Clearly define your research objectives, questions, and hypotheses

## Conclusions

Whilst crafting a research introduction may seem a challenging and time-consuming task, it is well worth the effort to convey your research clearly and engage potential readers. Providing sufficient background information on the research topic, conducting a comprehensive review of the existing research, determining the knowledge gap, understanding any limitations or controversies in the topic of interest, before then exploring any theoretical or conceptual frameworks to develop the research concepts, research questions and methodology are fundamental steps. Articulating any conceptual and operational definitions of key concepts and clearly defining any key terms, including explanations of how these will be used in the study is also paramount to a good introduction. It is essential to clearly present the rationale behind the research and why this is significant, clarifying what it adds to the existing body of knowledge in medical education and exploring any potential future implications. Lastly, it is vital to ensure that any research questions are clearly stated and are open-ended and exploratory in the case of qualitative studies, or specific and measurable in the case of quantitative studies.

We feel that observing these basic principles and adhering to these few simple steps will hopefully set the stage for a highly successful piece of research and will certainly go some way to achieving a favourable editorial outcome for possible subsequent publication of the work.

### Conflict of Interest

The authors declare that they have no conflict of interest.
